# A New Method of Evaluating the Symmetry of Movement Used to Assess the Gait of Patients after Unilateral Total Hip Replacement

**DOI:** 10.1155/2019/7863674

**Published:** 2019-12-01

**Authors:** Slawomir Winiarski, Alicja Rutkowska-Kucharska, Andrzej Pozowski, Krzysztof Aleksandrowicz

**Affiliations:** ^1^Biomechanics Department, University School of Physical Education in Wroclaw, Poland; ^2^Department of Physiotherapy, Faculty of Health Sciences, Wroclaw Medical University, Wroclaw, Poland

## Abstract

**Purpose:**

We propose a new concept of symmetry, the symmetry function, as a continuous function of the percentage of differences between sides of body movement and normalised throughout the whole range of motion. The method is used to assess the dynamical symmetry of gait of patients after unilateral total hip replacement (asymmetric group) and healthy people (symmetric group) and also to reveal discrepancies between normal and abnormal movement patterns.

**Methods:**

The gait of twelve male patients (49.7 ± 2.8 y), six weeks after unilateral total hip replacement (uTHR), was analysed against the gait of thirteen healthy men (36.1 ± 3.1 y). The speed of healthy men was matched to the speed of the patients. Comparison of the affected limb in uTHR patients with the healthy limb of able-bodied men was carried out on the basis of the highest symmetry values in the sagittal plane.

**Results:**

In uTHR patients, the symmetry function provides information on the symmetry of movements in the whole range of motion in contrast to symmetry indices which are calculated for selected parameters or peak values. Research revealed average asymmetric discrepancies for pelvic tilt up to 250% for the entire gait cycle with a peak of approx. 400% at the end of the loading response and terminal swing phases. Asymmetry of gait observed in other joints was below 200% of the mean range of motion.

**Conclusions:**

Regions of the greatest asymmetry in pathological movements are usually different from the region of the greatest range of motion. Therefore, it is insufficient to measure symmetry only for selected regions during motion. The symmetry function is a simple method which can complement other robust methods in time series data evaluation and interpretation.

## 1. Introduction

The symmetry of human movement is frequently understood as the perfect correspondence between the action of both lower limbs [1, 2]. The normal gait of a healthy adult is usually symmetrical, which reduces energy costs [3–6] and the risk of overloading joints [3, 7]. Several studies suggest that gait asymmetry may cause lower back pain (LBP) in people with motor disorders [8].

In clinical practice, the evaluation of symmetry, or more specifically, the degree of gait asymmetry, is achieved by assessing both the kinetic and kinematic parameters of gait. The determinants of normal gait are used to assess the normality of gait in the rehabilitation process. They are pelvic movement in the sagittal, frontal, and transverse planes and movements in the knee and ankle joints in the sagittal plane [9]. Others include the length and width of a stride and the symmetry of loading in the support phase. The degree of gait asymmetry of the determinants is important in functional restoration. There are many indices in functional diagnostics used to assess the degree of asymmetry; these have been used by many researchers and are as follows: symmetry index [2, 10, 11], relative difference index [12], relative asymmetry index [13], symmetry ratio [14], asymmetry ratio [15], integral index symmetry [16], symmetry angle [17], or the standard deviation of the differential index for upper limbs [18]. One of them, the symmetry index (SI), proved to be the most reliable tool for the assessment of side deviations in human movements and is widely used in movement analysis [19–21]. Unfortunately, the formula proposed originally by Robinson et al. [10] has some limitations, namely, SI being a single number, differences being compared against average values, and being ineffective overall for variables of small values.

In addition to index methods, more sophisticated tools that analyse time-dependent waveform traces are present and used as a data reduction tool or to determine the biomechanical features that distinguish pathological from normal pattern, e.g., factor analysis (FA) or principal component analysis (PCA) in parkinsonian gait [22–25], neuronal networks (ANN) in learning and classification of neuropathology [26–28], and statistical parametrical mapping (SPM) to analyse statistically subtle gait deviations [29–31]. These computer-based algorithms are typically performed after decorrelation and dimensionality reduction of data samples and thus can be laborious and slow on the large data sets, which makes them unsuitable in clinical settings. Many source separation algorithms optimise different criteria, and it remains subject to further research which method is the most robust for a specific data type.

Although studies evaluating the symmetry of normal and pathological gait have been conducted by many authors, there is still a need to measure deviation from the normal pattern in the temporal function of the gait cycle. Therefore, the aim of this study was to propose a new tool and concept, the symmetry function (SF), to evaluate the diversity of time series throughout the whole range of motion and to verify whether SF locates the regions of the greatest asymmetry in the entire gait cycle on the example of three cases, i.e., gait of patients after unilateral total hip replacement (uTHR) 6 weeks postoperation (asymmetrical by definition), gait of healthy people (symmetrical by definition), and gait of uTHR patients in comparison to normative values.

During walking, especially in the initial stage of physiotherapy, unilateral total hip replacement (uTHR) patients transfer the load to the unoperated limb to protect the operated one. This often leads to changes in the kinematics and kinetics of the uninvolved limb, which may be the cause of its overload but also underestimates the scores of pathology estimates. Therefore, it is reasonable to follow the movement parameters of both limbs separately and compare them with the norm of healthy people. This approach in assessing the improvement process—in our opinion—is more objective and applicative.

The evaluation of the diversity between time series should have an interpretation similar to the most commonly used symmetry indices. When comparing time series from different sources (between sides, subjects, and research groups), it should represent the percentage difference in relation to the average range of change in their value, therefore being normalised throughout the whole range of movement for a given joint and degree of freedom.

## 2. Method

### 2.1. Subjects

Twelve male patients (49.7 ± 2.8 years, 76.3 ± 9.1 kg, and 1.70 ± 0.16 m) after unilateral total hip replacement (uTHR group) and thirteen healthy, able-bodied men (normal group) (36.1 ± 3.1 years, 69.23 ± 9.1 kg, and 1.75 ± 0.15 m) took part in the experiment. The uTHR participants were measured 6 weeks postoperation in the period of the greatest stride asymmetries [32]. The normal group walked with low speed (1.16 ± 0.17 m/s) [33] to match the average walking speeds of the patients (0.96 ± 0.13 m/s) in the second physiotherapy examination stage [32]. The groups were speed-matched, because not age but rather the walking speed significantly influences the kinematic and kinetic variables of gait [34–37]. All participants were physically fit and engaged in different types of recreational activities. The exclusion criteria of the study were medical history of musculoskeletal injuries causing pain (other than uTHR), weakness, decreased range of motion, or loss of coordination and dysfunction of the neuromuscular, cardiovascular, or respiratory systems. They voluntarily participated and signed a consent form.

### 2.2. Procedure

The biomechanical assessment involved measuring spatiotemporal and angular gait variables using the BTS Smart-E motion analysis system. The BTS Smart-E motion analysis system was equipped with 6 digital near-infrared cameras (with a wavelength of 1.1 *μ*m light spectrum) at 120 Hz sampling frequency. A modified Helen Hayes hospital marker set was used in this study according to the ISB recommendation [38].

All markers were attached to the patients one by one by an experienced technician. Markers were placed to the right and left of the anterior superior iliac spine (ASIS) and the sacrum (midpoint between right and left posterior superior iliac spine, PSIS) defined by the pelvis. The thigh segments were defined by the hip-joint centre, and markers were placed on the femoral epicondyle and femoral wand, while calf segments were defined by the knee-joint centre and markers positioned on the malleolus and tibial wand. Foot segments were defined by markers positioned on the 2^nd^ metatarsal head, heel, and malleolus. This segment definition, along with the tracked data collected in the analysis, was required to calculate joint angles and segment orientations.

Subjects were asked to walk along a distance of 6 metres. Gait measurement for each subject was repeated 3 times, and each repetition contained 3 gait cycles. For both subject groups, the biomechanical assessment of each participant involved measuring spatiotemporal gait variables and the range of motion (ROM) in the main, lower extremity joints, especially those characterising the uTHR pattern in the sagittal plane:

- The pelvic tilt angle measured with respect to the global coordinate system as the angle between the horizontal pelvic plane and a line drawn from the ASIS to the PSIS; a positive value (up) corresponds to the normal situation in which the PSIS is higher than the ASIS
- The hip flexion-extension angle measured with respect to the pelvis coordinate system as the rotation of the proximal-distal axis about the mediolateral axis; a positive (flexion) angle value corresponds to the situation in which the knee is in front of the body
- The knee flexion-extension angle measured with respect to the femur coordinate system as the rotation of the proximal distal axis about the mediolateral axis; a positive angle corresponds to a flexed knee
- The ankle dorsi-plantar flexion angle measured with respect to the tibia coordinate system as the rotation of the proximal distal axis about the mediolateral axis; a positive number corresponds to dorsiflexion

**Figure 1:**
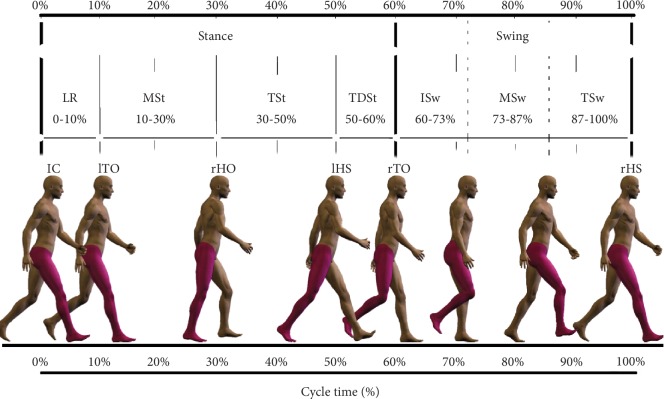
Gait terminology adopted in the analysis. Division of the gait cycle into phases: pelvic tilt (anterior/posterior), hip flexion/extension, knee flexion/extension, and ankle plantar flexion/dorsiflexion; sagittal view.

Gait cycle events as defined by Perry [39] were adopted for detailed descriptions: initial contact (IC, 0–2 of cycle time, %CT), load response (LR, 0–10%CT), midstance (MSt, 10–30%CT), terminal stance (TSt, 30–50%CT), terminal double stance (TDSt, 50–60%CT), initial swing (ISw, 60–73%CT), midswing (MSw, 73–87%CT), and terminal swing (TSw, 87–100%CT) (Figure 1).

All measurements were made in the certified Laboratory of Biomechanical Analysis of our university.

### 2.3. Symmetry Function

A variant of the symmetry index modified for temporal dependence was developed prior to this study. This symmetry function (SF) is a function of time and expresses the percentage difference between the tested right *x*_R_(*t*) and left *x*_L_(*t*) sides relative to the average range of motion (ROM).

(1) $$\begin{array}{c} \text{SF}\left(t\right)=\frac{x_{\text{R}}\left(t\right)-x_{\text{L}}\left(t\right)}{0.5\cdot \left[\text{Range}\left(x_{\text{R}}\left(t\right)\right)+\text{Range}\left(x_{\text{L}}\left(t\right)\right)\right]}\cdot 100\%. \end{array}$$

The positive/negative sign of the continuous scores indicates the leading side. A positive sign signifies that the first limb ranges more than the second. A score close to zero indicates symmetry (equality) between limbs and score of ±200% a hypothetical situation of comparing limbs of significant difference in range. Magnitudes of 15% or more in symmetry indices are often associated with subjects who have sustained an injury, whereas magnitudes below 10% are typically reported in noninjured populations [40]. Therefore, an asymmetry level of 10% or more is believed to place additional strain on the contralateral leg, compromising the subject's performance and predisposing to various injuries [41, 42].

For each participant and each of the measured cycles, time normalisation of the right and left angles for the sagittal plane was performed numerically by means of decomposition of a time series (trend detection) using Lagrange interpolating polynomial as a tool for curve fitting (Lagranges.m code). In this way, right and left cycles of the same length (100%CT) with discrete values (every 1%CT) were obtained.

In order to verify the methodology and to check the significance of found differences, the graphs of the range of motion in joints and SF were parameterised, i.e., for each graph, separately for the left and right sides, the highest and lowest values reached by the assigned angle-time characteristics during a test were extracted, including peak maximum (Peak^max^) and peak minimum (Peak^min^) values, range of changes in ROM in degrees (°), time to reach peak values and *t*_min_ and *t*_max_ as percent of time of gait cycle (%CT). Time parameters were used to localise areas of greatest asymmetry.

### 2.4. Statistical Analysis

All individual data from the unilateral total hip replacement (uTHR) and normal groups were subject to further analysis. The basic descriptive statistics (arithmetic means and standard deviations) were evaluated for the extracted values of Peak^min^, *t*_min_, Peak^max^, *t*_max_, and ROM. The Shapiro-Wilk test was used to test the normality of data distribution, and the parametric *t*-test was used to test the differences between sides (*α* = 0.05) and between uTHR and normal groups (*α* = 0.05).

## 3. Results

**Figure 2:**
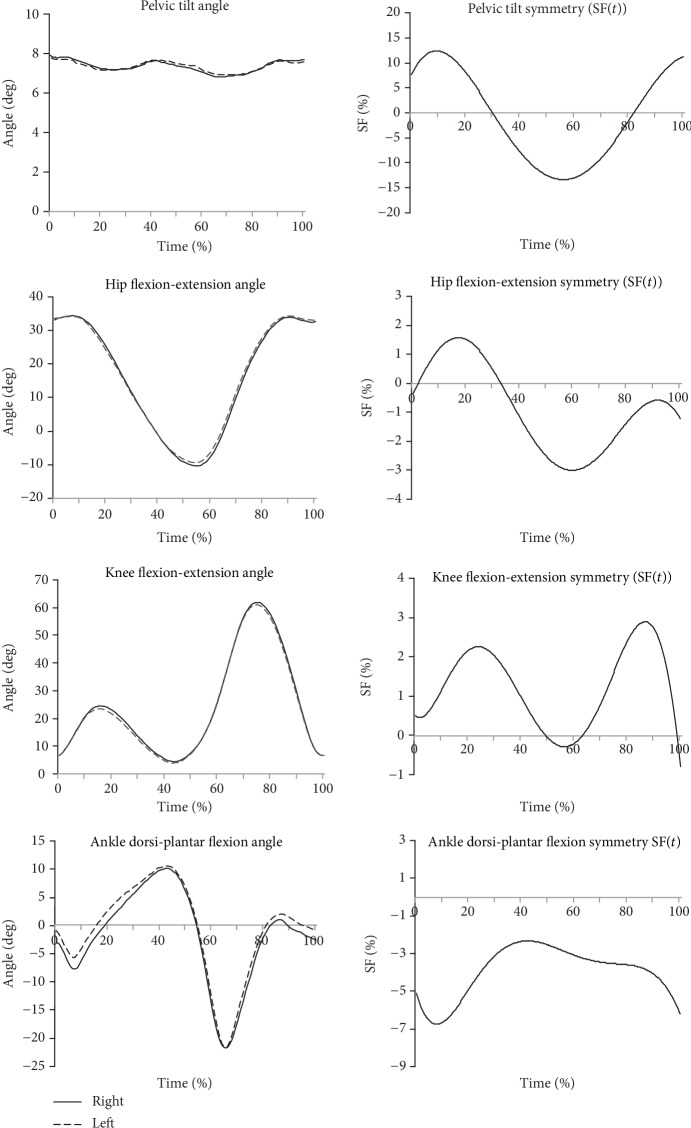
Angular joint kinematics in the sagittal plane and corresponding symmetry function (SF) for the normal group. Positive values of SF indicate right-side dominance in asymmetry, while negative values show left-side dominance.

**Figure 3:**
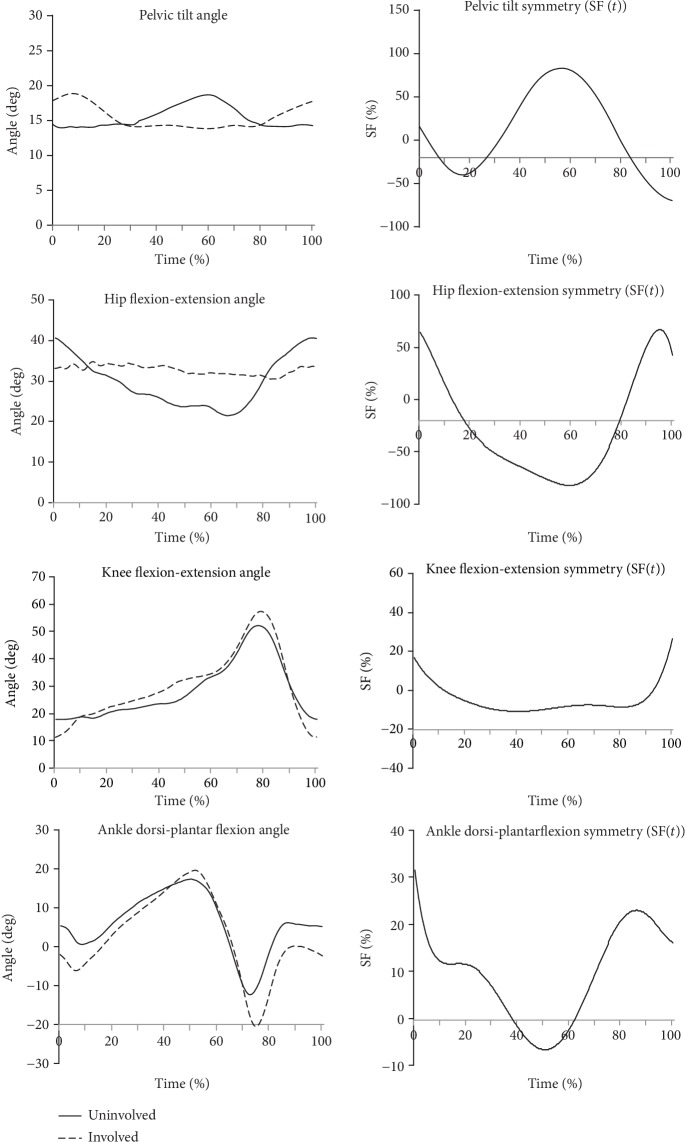
Angular joint kinematics in the sagittal plane and corresponding symmetry function (SF) for the uTHR group. Positive values of SF indicate uninvolved side dominance in asymmetry, while negative values show involved side dominance.

**Figure 4:**
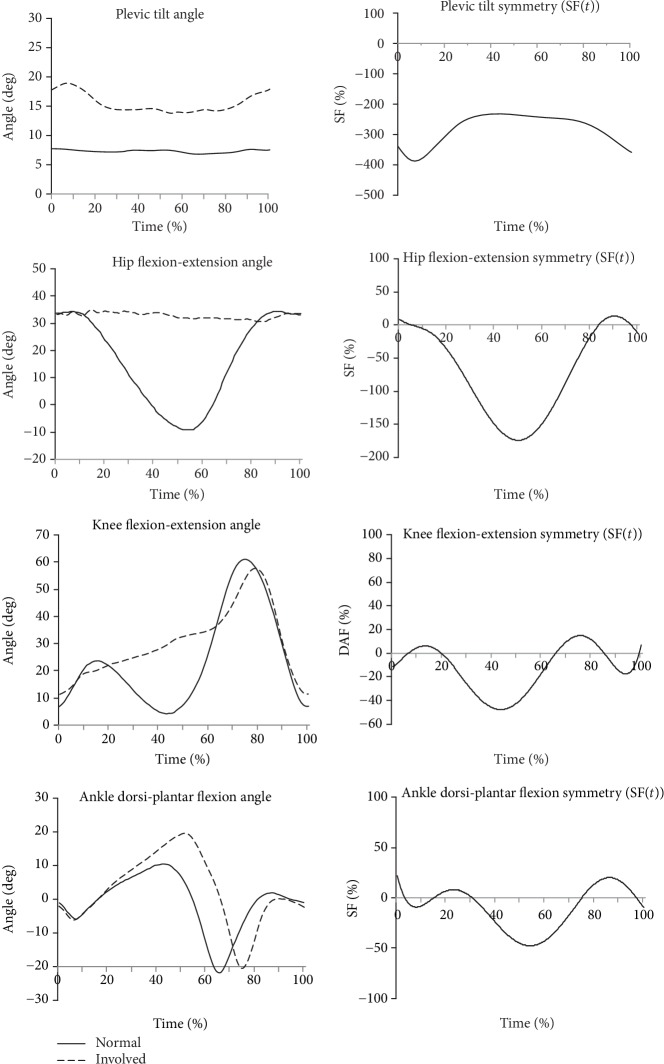
Angular joint kinematics in the sagittal plane and corresponding symmetry function (SF) for the involved limb of the uTHR group vs. normal subjects. Positive values of SF indicate normal curve dominance in asymmetry, while negative values show dominance of the curve for involved limb of the uTHR group.

Figures 2–4 show example results of the angle-time characteristics and corresponding symmetry function (SF) for the pelvic tilt, hip and knee flexion-extension, and ankle dorsi-plantar flexion angle for the normal subjects (Figure 2), for the uTHR patients (Figure 3), and the uTHR patients compared to normal subjects (Figure 4).

Positive values of SF indicate right-side dominance in asymmetry, while negative values show left-side dominance. Pelvic tilt asymmetry for the healthy group (Figure 2) changed by approx. 10% at the end of LR (30%CT) and TDSt (approx. 60%CT). Minor differences between the right and left sides for flexion-extension did not exceed 2%. The greatest, approx. 2% difference, was in MSt (20%CT) and approx. 3% at the end of TDS (approx. 60%CT). The greatest knee flexion-extension symmetry which did not exceed 5% was in MSt (approx. 15%CT) and in MSw (approx. 80%CT). Ankle dorsi-plantar flexion symmetry was negative at all times due to the higher mean values of the angle for the left side. The highest asymmetry of approx. 5.5% was in the LR to MSt (from 0 to 30%CT) at the end of the support phase in 60%CT and in the preswing phase (approx. 85%CT).

In uTHR patients (Figure 3), the difference between the sides was significantly higher. It was especially visible in the mean values of the SF. Symmetry for the angle of pelvic tilt was approx. 100% at the end of LR (10%CT) and TDSt (approx. 60%CT) phases. Hip flexion-extension symmetry was the highest (approx. 60%) at the beginning and end of the cycle and at the end of the support phase (approx. 100% asymmetry). Symmetry for the flexion-extension in the whole cycle did not exceed 20% and was the highest at the beginning and end of the gait cycle (approx. 18%). High asymmetry was also observed in a foot movement. The highest (approx. 23%) was in LR (10%CT), approx. 8% at the beginning of the ISw phase (60-70%CT) and 30% in MSw (80%CT).

The greatest differentiation was observed when the affected limbs of uTHR patients were compared with the healthy limbs of the normal subjects (Figure 4). A negative SF value indicates higher values of the angle for the affected limb and positive values show higher values of the normal limb. Pelvic tilt showed the greatest discrepancies between angular characteristics. Asymmetry for pelvic tilt was 250% for the entire gait cycle and hit a peak (approx. 400%) at the end of LR (10%CT) and the TSw (90-100%CT) phase. The highest values (over 150%) for hip flexion-extension symmetry were in TSw (50-60%CT), which resulted from the lack of ability to flex the hip joint. Angular values in the knee joint were not significantly different but still exceeded 50%. TSw (40-60%CT) showed the greatest asymmetry and was caused by the inability to extend the knee joint. Relatively large asymmetries reaching 50% were observed in the ankle joint at the end of the support phase (50-60%CT) and the beginning of the swing phase (60-70%CT).

Table 1 shows the exact SF values for differences between body sides for the range of motion (ROM) in the uTHR and normal groups. There was a statistically significant difference (*p* < 0.05) between sides in the majority of ROM parameters. There were no differences in ROM observed for pelvic tilt but a significant phase shift in the angular characteristic curve. Significant differences between the sides for the normal group were not reported. The SF values for the normal group usually did not exceed 5% of the mean ROM value (Figure 2, Table 2). Pelvic tilt was the exception. The differences between the sides were up to 12% of the average ROM. Angular characteristics for the uTHR patients measured 6 weeks postoperation differed significantly from those obtained for the healthy limb and normal group pattern (Figure 3). The highest SF values were recorded for pelvic tilt ROM (195.8 ± 5.59%) and hip flexion-extension ROM (108.9 ± 3.48%).

Table 2 presents peak values and ROM. Comparisons of the operated (uTHR) and healthy limb (normal group) showed the highest SF values (Figure 4). The highest SF(-) 386.7% for pelvic tilt was in approx. 8% cycle time (%CT) and remained at the average level of 300% throughout the movement. Asymmetry developed during hip joint flexion and extension resulted from limited ROM. The highest value of SF 146.2% occurred in approx. 54%CT, and the range of changes in SF reached 155.4% of the average ROM for this joint. Asymmetric movements were observed in knee and ankle joints. However, SF values were below 100% of the average ROM for the given degree of freedom.

The normal group showed less variation (standard deviation) than the uTHR group.

## 4. Discussion

Motion analysis plays an important role in clinical management of neurological and orthopaedic conditions. There has been growing interest in performing movement analysis in real time to provide instantaneous feedback to both analyst and patient. During the analysis, patients' movement profiles, as assessed by individual scalar or time series gait features, are compared against reference normal databases [43]. These actions are often reinforced by automated numerical algorithms of signal processing. Modern time series analysis comprises different robust methods for analysing and comparing series of data. There are two main approaches to the analysis: frequency and time domain. The first approach deals with the frequency domain to determine the subtle spectral components of series and to distinguish data trend from seasonal changes [44, 45]. The second approach represents time series as a function of time [46]. Time-domain techniques may be, additionally, divided into parametric and nonparametric methods. Parametric models are applied at each elementary signal component, using a general model, to describe the variability in the data in terms of experimental and confounding effects or residual variability. These methods are suitable to detect sets of identifiable data patterns, like rhythms, shifts, local pulses, and time trends [22–25, 29–31, 47]. Some researchers demonstrated that nonparametric machine learning techniques (e.g., nonparametric kernel estimation and polynomial regression) generally perform well due to their ability to capture the nondeterministic and complex nonlinearity of time series [48–50]. Particularly, artificial neural networks (ANN) in learning and classification of neuropathology have been successfully applied in gait analysis [26–28]. These numerical algorithms are typically performed through sophisticated data processing which is a laborious, time-consuming task that is subject to potential errors, particularly for large amounts of data [51].

Still, in clinical practice, simpler methods are used based on the analysis of symmetries in data parameters. Symmetry, as assessed by symmetry indices, is a measure of the differentiation and degree of pathology. It is accepted that normal walking should be symmetrical (i.e., the right limb does the same as the left limb but with a time lag). Side angular characteristics for healthy people are comparable and the degree of similarity generally does not exceed 10% [40, 52, 53] and depends on the analysed variable, joint, or degree of freedom [13, 19]. Symmetry for side angular characteristics in patients assessed continuously often exceeds 10% and asymmetric regions may persist throughout the movement [5, 21].

In this study, the dynamic symmetry function (SF) was employed to compare time courses and to locate regions of symmetry/asymmetry in the entire range of motion. In the analysis, the whole range of data points was taken into consideration. The highest values of SF were observed for movements in the operated joint. SF for pelvic tilt ROM and hip flexion-extension ROM often exceeded 100%, while for peak values (Peak^max^ and Peak^min^) for uTHR vs. normal this exceeded 250%. The greatest differences in angular characteristics increasing SF were the result of pelvic movement in the anterior tilt and limited range of motion in the hip joint of patients after uTHR. The region with the highest asymmetry did not coincide with the region of the greatest range of motion for most of the analysed joints. The comparison of uTHR and the normal group showed a correlation between the angle of hip flexion and knee extension. The highest hip flexion-extension SF (146.2%) value was in 54% of gait cycle (%GC), which coincided with *t*_min_ for hip flexion. The highest knee flexion-extension SF value was observed for the highest value of knee hyperextension.

There have been many studies that carried out on the symmetry of normal gait and various forms of pathological gait. However, there are very few tools for quantifying differences in time and deviations from the normal pattern. The symmetry indices used by researchers in the case of a small range of motion in a joint indicate significant asymmetry. This is due to the fact that small differences in the range of motion cause its increase. Moreover, measurement error in the case of such a small range significantly affects its value [2]. Symmetry indices are often used to calculate single values of symmetry for only extreme values in the time characteristics of joint movement. Such calculations seem unjustified due to the risk of underestimating the real values of asymmetry. The SF not only estimates symmetry values in the region of maximum value occurrence for which symmetry is most often assessed but also checks the proximity of these regions. The SF designates regions which are similar or not and indicates their degree of differentiation. Symmetry indices give only one value for the selected time in the entire gait cycle for maximum peak values of the angle. SF does not have these restrictions, i.e., it does not take infinite values near a singular point. It is precise (for both large and small values) and standardised. SF values represent the degree of similarity (symmetry) or differences (asymmetry) of the compared graphs, and the +/- indicates the side of asymmetry. The study has shown that SF differentiates the subjects, e.g., patients with single-limb injuries. It can be successfully applied to other movements, including other sports (gymnastics, synchronised swimming, etc.) where symmetry is important in evaluation. This method can assess the symmetry between the sides for time courses and the deviation of these courses from standard normative waveforms. SF is a time-dependent function describing symmetry in the duration of motion. It can be interpreted similarly to the SI and is relatively simple to calculate. SF is especially dedicated to time-dependent variables and corresponds well with the index proposed by Nigg et al. [54] for the stance phase. Used in assessing symmetry or differences in courses, SF calculates the relative difference to the average range of motion. It is normalised throughout the duration of movement for a given joint and degree of freedom and has a similar interpretation as the symmetry index for single values. However, the SF has a significant limitation. The SF is not normalised in relation to movements in adjacent joints, and its value depends on the average value of the range of the right and left body side. For instance, a similar difference in angular values Δ*α* = 1 deg for an average ROM = 5 deg (as in the case of pelvic tilt ROM for the uTHR group) gives SF = 20%, and for the average ROM = 50 deg (as in the case of knee flex-ext ROM for the normal group) gives SF = 2%. Therefore, when reporting the value of SF, it is recommended to specify the movement it has been calculated for or as supplementary information to give the absolute angle difference. The SF requires time courses of equal duration, but this is not a problem for modern gait analysis systems.

The method can be used to locate regions with the highest asymmetry in the gait cycle in patients with unilateral impairments. The surgical or physiotherapeutic intervention does not solve the problem of the gait asymmetry perpetuated by time. Functional restoration carried out by a physiotherapist significantly reduces the degree of asymmetry. Their work can be supported by the use of the symmetry function to evaluate the effects of physiotherapy. Moreover, this simple method of time series data comparison can complement other robust methods in data evaluation and interpretation.

## 5. Conclusions

- The symmetry function (SF) is relevant to the tested variable of the same test group or to determine discrepancies in test parameters between pathological and control group
- SF provides information on the symmetry of movements in the whole range of motion in contrast to symmetry indices which are calculated for selected parameters or peak ROM values
- The SF is normalised throughout the duration of movement for a given joint and degree of freedom and has a similar interpretation as the symmetry index for single values
- Regions of the greatest asymmetry in pathological movements are usually different from the region of the greatest range of motion. Therefore, it is insufficient to measure symmetry only for selected regions during motion
- The SF is not standardised in relation to movement in adjacent joints. Thus, when reporting the value of SF, it is recommended to indicate which movement it has been calculated for or to specify the absolute difference in angle

## Figures and Tables

**Table 1:** Mean ± standard deviation for the range of motion (ROM) for the uTHR and normal groups.

	uTHR		Normal	
	Involved	Uninvolved	Left	Right
*Pelvic tilt angle*				
Peak^min^ (°)	14.0 ± 0.69^#^	13.9 ± 0.46^#^	6.9 ± 0.22	6.8 ± 0.32
*t*_min_ (%GC)	61.0 ± 0.1^∗^^#^	8.0 ± 0.1^#^	70.0 ± 3.7	69.0 ± 0.9
Peak^max^ (°)	18.9 ± 0.77^#^	18.6 ± 0.41^#^	7.8 ± 0.30	7.9 ± 0.25
*t*_max_ (%GC)	7.0 ± 0.2^∗^^#^	58.0 ± 0.9^#^	0.9 ± 0.4	0.8 ± 0.8
ROM (°)	4.9 ± 0.25^#^	4.7 ± 0.07^#^	0.9 ± 0.23	1.1 ± 0.11
*Hip flexion-extension angle*				
Peak^min^ (°)	30.5 ± 0.40^∗^^#^	21.5 ± 0.77^#^	−9.5 ± 0.46	−10.4 ± 0.42
*t*_min_ (%GC)	82.0 ± 0.5^#∗^	66.0 ± 3.1^#^	53.0 ± 1.9	54.0 ± 2.6
Peak^max^ (°)	43.1 ± 1.03^#^	40.5 ± 0.41^#^	34.3 ± 1.37	34.6 ± 0.93
*t*_max_ (%GC)	0.8 ± 1.0^#^	0.2 ± 0.9^#^	89.0 ± 4.0	92.0 ± 1.3
ROM (°)	12.6 ± 1.29^∗^^#^	19.0 ± 0.61^#^	43.5 ± 1.83	45.0 ± 1.98
*Knee flexion-extension angle*				
Peak^min^ (°)	11.4 ± 0.34^∗^^#^	17.8 ± 0.41^#^	4.0 ± 0.14	4.5 ± 0.16
*t*_min_ (%GC)	0.3 ± 0.7^#^	0.6 ± 0.5^#^	43.0 ± 0.1	43.0 ± 0.9
Peak^max^ (°)	57.5 ± 2.88^∗^	52.0 ± 1.14^#^	60.9 ± 2.50	61.8 ± 2.22
*t*_max_ (%GC)	79.0 ± 0.7^#^	77.0 ± 2.8	74.0 ± 2.1	74.0 ± 2.8
ROM (°)	46.1 ± 1.24^∗^^#^	34.2 ± 0.92^#^	56.9 ± 0.57	57.3 ± 1.09
*Ankle dorsi-plantar flexion angle*				
Peak^min^ (°)	−20.4 ± 0.73^∗^	−12.2 ± 0.37^#^	−21.6 ± 0.41	−21.8 ± 1.05
*t*_min_ (%GC)	75.0 ± 2.0^#^	72.0 ± 1.8^#^	65.0 ± 1.2	65.0 ± 2.9
Peak^max^ (°)	19.6 ± 0.71^∗^^#^	17.4 ± 0.19^#^	10.5 ± 0.50	10.1 ± 0.04
*t*_max_ (%GC)	51.0 ± 0.5^#^	50.0 ± 1.7^#^	42.0 ± 2.0	42.0 ± 1.8
ROM (°)	40.0 ± 1.68^∗^^#^	29.6 ± 1.39	32.1 ± 0.88	31.9 ± 0.61

**Table 2:** Mean ± standard deviation SF for the uTHR and normal groups and between the uTHR and the right normal limb.

	uTHR	Normal	uTHR vs. normal
*Pelvic tilt*			
Peak^min^ (°)	−102.1 ± 1.33^∗^^#^	−11.6 ± 4.24^#^	−386.7 ± 18.56
*t*_min_ (%GC)	8.0 ± 0.7^∗^	58.0 ± 0.8^#^	8.3 ± 0.2
Peak^max^ (°)	93.8 ± 3.19^∗^^#^	10.1 ± 0.32^#^	−232.1 ± 11.14
*t*_max_ (%GC)	60.0 ± 0.8^∗^^#^	11.0 ± 0.1^#^	55.0 ± 0.5
ROM (°)	195.8 ± 5.59^∗^^#^	30.9 ± 7.74^#^	154.6 ± 5.41
*Hip flexion-extension*			
Peak^min^ (°)	−64.6 ± 2.39^∗^^#^	−3.2 ± 2.01^#^	−146.2 ± 2.05
*t*_min_ (%GC)	66.0 ± 2.6^#^	60.0 ± 2.4	54.0 ± 3.3
Peak^max^ (°)	44.3 ± 2.13^∗^^#^	2.2 ± 0.09^#^	9.3 ± 0.30
*t*_max_ (%GC)	99.6 ± 3.4^∗^^#^	20.0 ± 0.3^#^	87.0 ± 4.4
ROM (°)	108.9 ± 3.48^∗^^#^	5.3 ± 0.14^#^	155.4 ± 3.11
*Knee flexion-extension*			
Peak^min^ (°)	−17.4 ± 0.26^∗^^#^	−0.8 ± 0.02^#^	−51.7 ± 2.27
*t*_min_ (%GC)	47.0 ± 1.7^∗^	59.0 ± 1.6^#^	46.0 ± 0.8
Peak^max^ (°)	15.9 ± 0.64^∗^^#^	3.1 ± 0.04^#^	22.4 ± 0.29
*t*_max_ (%GC)	0.9 ± 2.7^∗^^#^	85.0 ± 1.4^#^	70.0 ± 1.3
ROM (°)	33.3 ± 0.67^∗^^#^	3.9 ± 0.11^#^	74.0 ± 0.67
*Ankle dorsi-plantar flexion*			
Peak^min^ (°)	−7.3 ± 2.16^#^	−6.7 ± 3.07^#^	−69.4 ± 1.74
*t*_min_ (%GC)	52.0 ± 1.5^∗^^#^	7.0 ± 0.0^#^	63.0 ± 3.0
Peak^max^ (°)	31.4 ± 2.13^∗^^#^	−0.6 ± 1.23^#^	37.8 ± 1.78
*t*_max_ (%GC)	78.0 ± 2.9^∗^	66.0 ± 1.6^#^	76.0 ± 1.1
ROM (°)	38.7 ± 1.74^∗^^#^	6.0 ± 0.18^#^	107.3 ± 0.32

## Data Availability

All data (individual results and values behind the means for all measures reported) used to support the findings of this study are included within the article or are available from the corresponding author upon request.
